# Enteral Nutrition Versus Parenteral Nutrition on Outcomes in Acute Pancreatitis: Insights From the Nationwide Inpatient Sample

**DOI:** 10.7759/cureus.44957

**Published:** 2023-09-09

**Authors:** Fidelis Uwumiro, Oluwatobi A Olaomi, Opeyemi Tobalesi, Victory Okpujie, Olawale Abesin, Enomen Ekata, Pascal Ezerioha, Uwakmfonabasi A Umoudoh, Zainab Olapade, Evaristus Asobara

**Affiliations:** 1 Internal Medicine, Our Lady of Apostles Hospital, Akwanga, NGA; 2 Radiology, University of Ibadan, Ibadan, NGA; 3 Internal Medicine, College of Health Sciences, University of Ilorin, Ilorin, NGA; 4 Internal Medicine, Central Hospital Benin, Benin City, NGA; 5 Internal Medicine, Royal Cornwall Hospitals NHS Trust, Cornwall, GBR; 6 Internal Medicine, Ambrose Alli University, Ekpoma, NGA; 7 Internal Medicine, Nnamdi Azikiwe University, Awka, NGA; 8 Internal Medicine, Southport and Formby District General Hospital, Southport, GBR; 9 Clinical Research, Lamar University, Beaumont, USA

**Keywords:** nationwide inpatient sample, pancreas, parenteral nutrition, enteral feeding, acute pancreatitis

## Abstract

Introduction: Despite considerable research on the comparison of enteral and parenteral nutrition in patients with acute pancreatitis, there is an ongoing debate about the optimal timing of nutrition initiation, invasiveness of interventions, impact on outcomes, and patient tolerance. Given the gap that still exists in the literature, we investigated the relationship between the mode of nutrition and critical outcomes such as mortality rates, inpatient complications, length of hospitalization, and discharge disposition, using comprehensive national-level data. In addition, we investigated the impact of early enteral nutrition on outcomes in acute pancreatitis.

Methods: All adult discharges for acute pancreatitis between 2016 and 2018 were analyzed from the National (Nationwide) Inpatient Sample (NIS). Discharges of minors and those involving mixed nutrition were excluded from the analysis. Enteral nutrition and parenteral nutrition subgroups were identified using the International Classification of Diseases, 10th revision (ICD-10) codes. Disease severity was defined using the 2013 revised Atlanta Classification of Acute Pancreatitis, along with the All Patient Refined Diagnosis Related Group (APR-DRG)'s severity of illness and likelihood of mortality variables. Complications were identified using ICD-10 codes from the secondary diagnoses variables within the NIS dataset. Multivariable logistic regression analyses were employed to assess associations between the mode of nutrition and the outcomes of interest.

Results: A total of 379,410 hospitalizations were studied. About 2,011 (0.53%) received enteral nutrition, while 4,174 (1.1%) received parenteral nutrition. The mean age of the study was 51.7 years (SD 0.1). About 2,280 mortalities were recorded in the study. After adjustments, enteral nutrition was associated with significantly lower odds of mortality (adjusted OR (aOR): 0.833; 95%CI: 0.497-0.933; P<0.001). Parenteral nutrition was linked with significantly greater odds of mortality (aOR: 6.957; 95%CI: 4.730-10.233; P<0.001). Both enteral nutrition and parenteral nutrition were associated with augmented odds of complications and prolonged hospitalization (P<0.001) compared to normal oral feeding. Initiation of enteral nutrition within 24 hours of admission did not improve the odds of mortality in this study (aOR: 5.619; 95%CI: 1.900-16.615; P=0.002).

Conclusion: Enteral nutrition demonstrates better outcomes in mortality rates and systemic complications compared to parenteral nutrition in patients unable to maintain normal oral feeding.

## Introduction

Acute pancreatitis (AP) is a common cause of hospitalization, with the literature reporting over 300,000 annual hospital admissions [1,2]. Recent studies indicate that the incidence of AP has increased over time, particularly in North America and Europe, with an average annual percent increase of 3.1% from 1961 to 2016 [3,4]. AP is a serious condition associated with high morbidity and mortality rates. Up to 20% of patients may develop severe AP, which can result in multi-organ failure, infections, and death [5,6].

The management of AP is supportive [7], aiming to relieve pain, prevent complications, and optimize nutrition [8,9]. Nutritional support is important in AP patients, especially in severe cases where the inflammatory response can lead to a catabolic state and malnutrition [10]. Various modes of nutritional support, including enteral nutrition (EN), parenteral nutrition (PN), and mixed nutrition, have been used in AP patients. EN is often preferred because it preserves gut barrier function and is the first-line option for nutritional support. PN is often used when EN is not possible or contraindicated. However, the optimal mode of nutrition in AP patients is still debatable, and the choice of nutritional support depends on the patient's clinical status, the severity of pancreatitis, and the presence of complications. Recent research suggests that EN may be more effective than PN in reducing infectious complications, improving gut barrier function, preventing bacterial translocation, and promoting earlier recovery in patients with AP [11-13]. However, other studies highlight the lack of sufficient data to conclusively establish the effectiveness or superiority of either mode of nutrition in this patient population [14].

This study aims to investigate the association between the mode of nutrition and the odds of mortality, length of hospital stay, complications, and discharge disposition (routine home discharge, discharge to nursing homes or home health care) using national-level data for patients admitted with AP. We hypothesize that EN will demonstrate better outcomes compared to PN.

## Materials and methods

Design and data source

We utilized the 2016-2018 National (Nationwide) Inpatient Sample (NIS) in this study. The NIS is the largest publicly available all-payer inpatient care database in the United States, containing information on over 21 million hospitalizations (7-8 million hospitalizations in each year of the NIS sample). The database rigorously reflects a 20% sample of all United States hospital admissions, excluding admissions to rehabilitation and federal hospitals (e.g., Veterans Affairs hospitals). The NIS sample includes data from all participating states (46 states and the District of Columbia), covering 98% of the United States population. Each year's NIS data contains around 7-8 million records, each with a corresponding discharge weight. Developed and maintained by the Agency for Healthcare Research and Quality (AHRQ) through the Healthcare Costs and Utilization Project (HCUP), the NIS's large sample size makes it well-suited for generating national estimates and analyzing rare diseases, uncommon procedures, and specific patient populations. Additionally, the NIS's self-weighting design reduces the margin of error and enables precise estimates to be produced [15,16].

Study population

**Figure 1:**
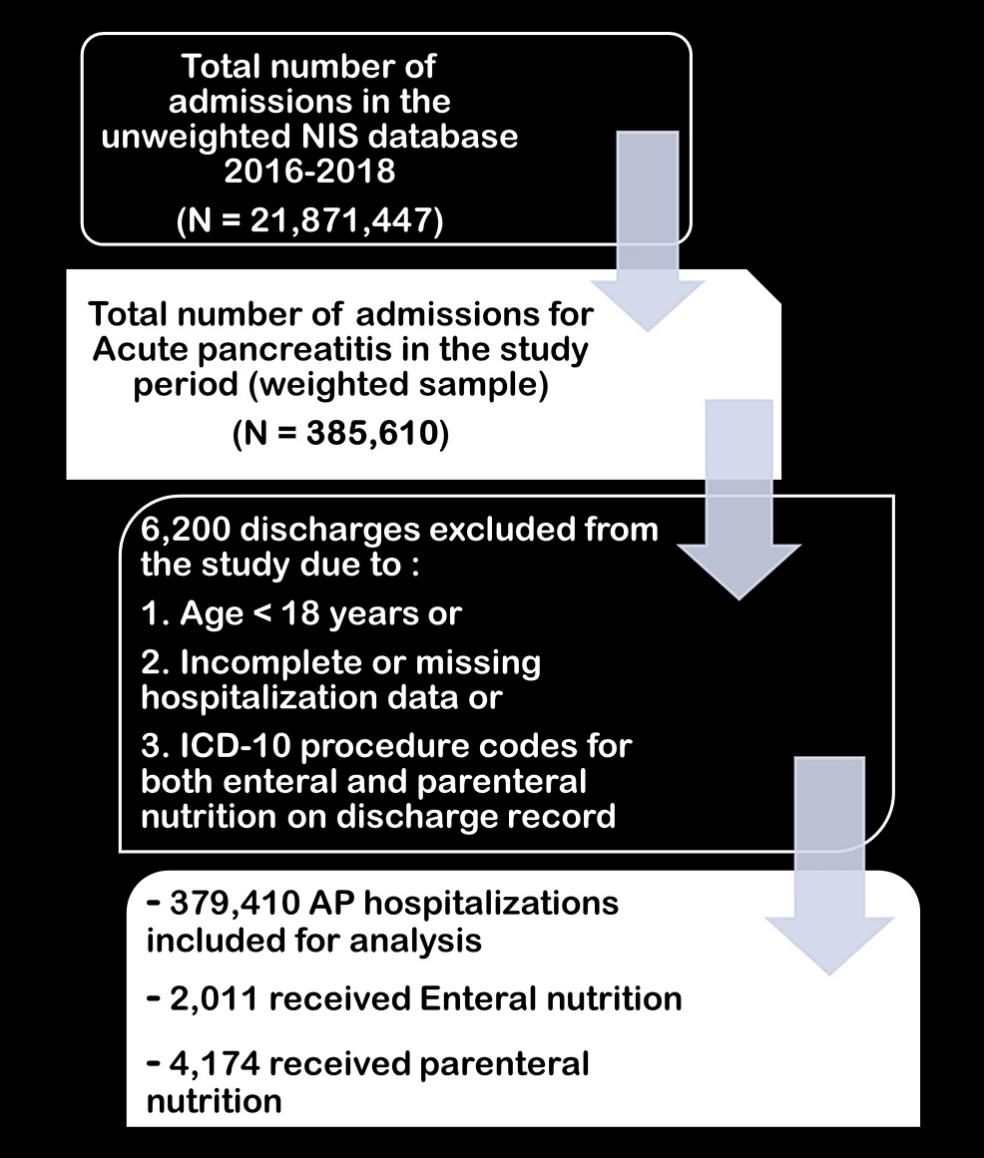
Study selection criteria NIS, National (Nationwide) Inpatient Sample; AP, acute pancreatitis; ICD-10, International Classification of Diseases, 10th revision

The study analyzed all discharge records for adults with a principal diagnosis of AP (International Classification of Diseases, 10th revision (ICD-10) code K85) from the NIS database [17]. The hospitalizations included in the study were classified into distinct sub-populations of AP based on severity, namely mild, moderately severe, and severe AP, using the 2013 Revised Atlanta Classification [18]. The EN and PN subgroups were identified using the ICD-10-Clinical Modification/Procedure Coding System (ICD-10-CM/PCS; appendix). Hospitalizations for patients under the age of 18, or who received mixed nutrition were excluded from the study (Figure 1).

Study variables

The NIS database contains pre-defined variables, including mortality, length of hospital stay (LOS), and primary discharge status. EN, PN, confounders, and complication variables, such as acute kidney failure, pancreatic pseudocyst/cyst, acute respiratory distress syndrome (ARDS), acute lung injury (ALI) with pleural effusion, sepsis, persistent hyperglycemia, pericardial effusion, pneumonia, altered mental status, splenic vein thrombosis, need for vasopressors, abdominal compartment syndrome, and disseminated intravascular coagulopathy were identified through ICD-10-CM PCS codes (See Appendix) and validated by published literature [19-21]. The number of days between a patient's admission and a procedure is recorded in the NIS. Thus, if a patient underwent EN or PN within 24 hours of admission, the time to the procedure was indicated as 0 or 1 day. According to the recommendations of the American College of Gastroenterology (ACG), as revised in 2018, patients with mild AP who do not exhibit symptoms of nausea and vomiting should be promptly started on normal oral feeding [22,23]. For the purpose of the index study, normal oral feeding was defined as the absence of EN or PN codes, the absence of ICD-10 codes for nausea and vomiting (R110, R1110, and R112), and APR-DRG severity and mortality levels of minor loss of function or minor likelihood of dying in the index hospitalization. Prolonged LOS was defined as AP-specific LOS in the top decile (15 days or longer). Non-home discharge was defined as all discharges other than routine home discharge. Any procedure/surgery was defined as the presence of ICD-10 codes for endoscopic retrograde cholangiopancreatography (ERCP) or pancreatic resection (Appendix).

Main outcomes

The primary outcome was in-hospital mortality. Secondary outcomes were inpatient complications, mean LOS, and patient discharge disposition (home vs. non-home discharge) based on the mode of nutritional support.

Statistical analysis

The data analysis was conducted using Stata Statistical Software: Release 17 (2021; StataCorp LLC, College Station, Texas, United States) in accordance with HCUP regulations. Weighted samples were applied to provide national estimates. Comorbidities were computed as proportions, and the χ2 test was employed to compare sociodemographic characteristics among the different patient subpopulations. To adjust for the burden of comorbidity and AP severity, we utilized the grouped Charlson Comorbidity Index (CCI) and the All Patient Refined Diagnosis Related Group (APR-DRG) respectively. Multivariable logistic regression analysis was performed to account for potential confounding variables, such as age, gender, CCI, APR-DRG, race or ethnicity, any complications, any surgeries, hospital location (rural or urban), geographic region (Northeast, Midwest, West, or South), hospital bed capacity, and hospital teaching status. A statistical significance threshold of p<0.05 was maintained to determine the outcomes. Frequencies were presented as absolute numbers and proportions of the study cohort while results of regression analyses were presented as crude odds ratio (OR) or adjusted odds ratio (aOR).

Ethical considerations

This study was conducted using de-identified data from the NIS database, which complies with the Health Insurance Portability and Accountability Act (HIPAA) of 1996 and lacks patient-specific and hospital-level identifiers. As a limited dataset, NIS does not require review by an institutional review board. Therefore, this study was exempt from Institutional Review Board approval.

Data availability statement

We used the 2016-2018 NIS database for this study. The NIS is the largest publicly available, all-payer inpatient care database containing data on more than seven million US hospital stays annually. NIS data is available online through the HCUP central distributor at http://www.hcup-us.ahrq.gov.

## Results

Study population and patient characteristics

The study analyzed 379,410 adult hospitalizations for AP. Among them, 178,045 were for mild AP, 119,580 were for moderately severe AP, and 81,785 were for severe AP. About 2,011 (0.53%) received EN, while 4,174 (1.1%) were administered PN during the index hospitalization. Table 1 presents an overview of the sociodemographic attributes of the study population stratified by the mode of nutrition recorded during the admission. The mean age of the total study cohort was 51.7 years (SD 0.1), and the majority of the total, parenteral, and enteral subpopulations were male (53.4%, 58%, and 56.7%, respectively). During the study period, there were more hospital admissions for pancreatitis among the White race than all other ethnic groups combined. Approximately 90.6% of the total hospitalizations were covered by Medicare, Medicaid, or private insurance, including health maintenance organization (HMO). The majority of the hospitalizations during the study period occurred in large teaching hospitals in the Southern United States, mainly for individuals in the 0-25th median annual income quartile.

**Table 1:** Socio-biodemographic properties of the study population stratified by mode of nutrition * The APR-DRG subclasses are employed by major United States hospital systems. These subclasses are derived from diagnosis and surgical procedure codes obtained through chart abstraction. The APR-DRG algorithm generates a DRG along with two modifiers: Severity of Illness (SOI) and Risk of Mortality (ROM). SOI indicates the extent of organ system loss or physiological decompensation and is divided into minor, moderate, major, and extreme categories. ROM estimates the likelihood of in-hospital mortality based on various factors. SOI and ROM are independent estimators of disease burden. a Sundararajan's adaptation of the modified Deyo's Charlson Comorbidity Index (CCI), which offers a refined approach for population-based investigations. This adaptation classifies the CCI into four distinct groups, each indicative of escalating mortality risk. A CCI score surpassing 3 is associated with an approximate 25% 10-year mortality rate, whereas scores of 2 or 1 correspond to 10% and 4% 10-year mortality rates, respectively. SD, standard deviation; LOF, loss of function; LOD, likelihood of dying; USD, United States dollar; HMO, health maintenance organization; COPD: chronic obstructive pulmonary disease; APR-DRG, All Patient Refined-Diagnosis Related Group

Patient and hospital variables	Enteral nutrition (n = 2,011)	Parenteral nutrition (n = 4,174)	P-value
Sex, n (%)			0.060
Male	1,166 (58.00)	2,367 (56.70)	
Female	844 (42.00)	1,807 (43.30)	
Race/ethnicity, n (%)			0.022
White	1,501 (74.67)	2,861 (68.54)	
Black	244 (12.14)	548 (13.12)	
Hispanic	177 (8.18)	478 (11.46)	
Asian or Pacific Islander	32 (1.58)	96 (2.29)	
Native American	21 (1.06)	37 (0.89)	
Other	48 (2.37)	154 (3.69)	
Mean age, (years ± SD)	50.2 ± 0.9	52 ± 0.1	0.020
Charlson comorbidity index score, n (%)			<0.0001
0	638 (31.75)	1,504 (36.04)	
1	608 (30.25)	1,073(25.71)	
2	352 (17.50)	667 (15.99)	
≥ 3	412 (20.50)	929 (22.26)	
APR DRG: severity of illness*, n (%)			<0.0001
Minor LOF	85 (4.25)	250 (6.00)	
Moderate LOF	351 (17.50)	789 (18.91)	
Major LOF	710 (35.30)	1,716 (41.10)	
Extreme LOF	865 (43.00)	1,419 (34.00)	
APR DRG: risk of mortality*, n (%)			<0.0001
Minor LOD	428 (21.30)	1,022 (24.50)	
Moderate LOD	492 (24.50)	1,031 (24.70)	
Major LOD	573 (28.50)	1,152 (27.60)	
Extreme LOD	518 (25.80)	973 (23.30)	
Comorbidities^a^, n (%)			
Uncomplicated diabetes	437 (21.75)	831 (19.93)	0.910
Chronic Liver Disease	407 (20.25)	929 (22.26)	0.440
COPD	377 (18.75)	667 (15.99)	0.113
Chronic renal disease	206 (10.25)	447 (10.70)	0.801
Complicated diabetes	126 (6.25)	31 (0.74)	0.040
Congestive heart failure	201 (10.00)	359 (8.61)	0.493
Acute myocardial infarction	75 (35.00)	185 (4.43)	0.453
Peripheral vascular disease	80 (4.00)	195 (4.67)	0.291
Cancer	65 (3.25)	226 (5.42)	0.140
Rheumatoid disease	65 (3.25)	118 (2.83)	0.184
Peptic ulcer disease	80 (4.00)	123 (2.95)	0.311
Dementia	50 (2.50)	81 (2.09)	0.103
Cerebrovascular disease	55 (2.75)	56 (1.35)	0.433
AIDS	10 (0.50)	31 (0.74)	0.010
Paralysis	40 (2.00)	31 (0.74)	0.541
Median annual income (quartiles) in patient’s zip code, n (%)			0.034
First (0-25th)	541(26.91)	1,128 (27.02)	
Second (26th– 50th)	526 (26.14)	1,097 (26.28)	
Third (51st-75th)	587 (29.19)	1,045 (25.03)	
Fourth (76th-100th)	357 (17.77)	905 (21.67)	
Insurance type			<0.0001
Private including HMO	797 (39.63)	1,637 (39.23)	
Medicare	628 (31.23)	1,247(29.87)	
Medicaid	507 (25.20)	1,032 (24.74)	
Uninsured	79 (3.94)	1,246 (6.15)	
Hospital region, n (%)			<0.0001
South	472 (23.51)	1,587 (38.01)	
Midwest	699 (34.75)	883 (21.16)	
West	357 (17.75)	909 (21.77)	
Northeast	482 (24.00)	796 (19.07)	
Hospital bed size, n (%)			<0.0001
Large	1,161 (57.75)	2,248 (53.87)	
Medium	618 (30.75)	1,303 (31.24)	
Small	231 (11.51)	621 (14.88)	
Weekend admission, n (%)	432 (21.5)	1,180 (28.29)	0.032
Hospital location/teaching status, n (%)			<0.0001
Urban teaching	1,654 (82.25)	2,865 (68.63)	
Urban non-teaching	297 (14.75)	1,053 (25.22)	
Rural	60 (3.00)	257 (6.15)	

Mortality

The study recorded a total of 2,280 mortalities. Among the cohort, 2,025 patients were admitted with severe pancreatitis, 169 with moderately severe pancreatitis, and 86 with mild pancreatitis. 359 individuals (8.6%) who received PN and 102 (4.5%) who received EN died in the index hospitalization.

The crude OR of mortality based on the mode of nutrition, were 6.957 (95%CI: 4.730-10.233; P<0.001) for EN and 9.149 (95%CI: 5.593-14.966; P<0.001) for PN. After adjusting for potential confounders including disease severity and comorbidity burden, EN was associated with lower odds of mortality (aOR: 0.833; 95%CI: 0.497-0.933; P<0.001), while PN was linked to an increased likelihood of mortality (aOR: 1.687; 95%CI: 1.045-2.722; P = 0.032). Notably, EN did not show any significant difference in mortality in cases of severe AP (aOR: 1.227; 95%CI: 0.982-1.493; P=0.57). Additionally, the study identified several independent factors associated with mortality, including patient age, the CCI, prolonged hospitalization duration, major loss of function in APR-DRG illness severity class, major likelihood of dying based on APR-DRG risk of mortality class, and the incidence of any complication during the index admission (Table 2).

**Table 2:** Logistic regression analysis of significant predictors of mortality on univariable and multivariable analyses ^a ^Defined as hospital admission lasting 15 or more days or in the top decile of the total study population. * Evidence of endoscopic retrograde cholangiopancreatography or resection of pancreas on discharge record. aOR, adjusted odds ratio; OR, unadjusted odds ratio; CI, confidence interval; LOF, loss of function; LOD, likelihood of dying; APR-DRG, All Patient Refined-Diagnosis Related Group

Variables	Univariable analysis		Multivariable analysis	
	p-value	aOR (95% CI)	p-value	aOR (95% CI)
Parenteral nutrition	<0.0001	6.957 (4.730-10.233)	0.032	1.687 (1.045-2.722)
Enteral nutrition	<0.0001	9.149 (5.593-14.966)	0.004	0.833 (0.497-0.933)
Age	<0.0001	1.050 (1.044–1.056)	<0.0001	1.048 (1.034-1.063)
Female Sex	0.040	0.818 (0.677–0.987)	0.102	0.844 (0.690-1.034)
Black race	0.036	0.750 (0.570-0.981)	0.198	0.827 (0.619-1.104)
Hispanic descent	0.016	0.681 (0.498-0.932)	0.624	0.921 (0.661-1.282)
Admission to large hospitals	0.002	1.517 (1.170-1.966)	0.288	1.160 (0.882-1.525)
APR-DRG: illness severity class				
Minor LOF	Reference	Reference	Reference	Reference
Moderate LOF	<0.0001	0.52 (0.326-0.820)	0.262	0.60 (0.251-1.455)
Major LOF	<0.0001	3.53 (2.779-4.478)	<0.0001	2.97 (2.163-4.084)
APR-DRG: risk of mortality				
Minor LOD	Reference	Reference	Reference	Reference
Moderate LOD	<0.0001	0.13 (0.067-0.238)	<0.0001	1.634 (0.812-3.286)
Major LOD	<0.0001	0.41 (0.239-0.932)	<0.0001	10.4 (7.400-14.677)
Charlson comorbidity index	<0.0001	1.407 (1.365–1.450)	<0.0001	1.198 (1.145-1.253)
Uninsured	0.002	0.507 (0.333–0.773)	0.240	1.361 (0.813-2.280)
Prolonged hospital stay^a^	<0.0001	9.081 (7.487–11.015)	<0.0001	3.236 (2.564-4.082)
Any complication	<0.0001	23.209 (17.205–31.309)	<0.0001	12.641 (9.121-17.519)
Any procedure/surgery*	0.085	1.525 (0.944-2.463)	0.532	0.842 (0.496-1.428)

Incidence and odds of inpatient complications

The overall incidence of complications was 26.7%. Hospitalizations with EN had complications in 53% of cases, while hospitalizations with PN had complications in 34.4% of cases. The most frequent complications observed were acute kidney failure, pancreatic cysts, acute respiratory distress syndrome, acute lung injury with pleural effusion, sepsis, persistent hyperglycemia, pneumonia, and pericardial effusion (Table 3).

**Table 3:** Incidence of complications recorded in the study ARDS, acute respiratory distress syndrome; DIC, disseminated intravascular coagulopathy; SIRS, systemic inflammatory response syndrome; ALI, acute lung injury

Complications	Total study population (n = 379,410)	Enteral nutrition (n = 2,011)	Parenteral nutrition (n = 4,174)
Acute kidney failure	47,388 (12.49)	542 (27.01)	1,165 (27.92)
Pancreatic pseudocyst/ cyst	27,545 (7.26)	462 (23.00)	1,047 (25.09)
ARDS	20,374 (5.37)	663 (33.00)	1,222 (29.27)
ALI with pleural effusion	18,211 (4.80)	708 (35.25)	1,242 (29.76)
SIRS/Sepsis	13,317 (3.51)	372 (18.5)	611 (14.64)
Persistent hyperglycemia	10,016 (2.64)	141 (7.01)	385 (9.23)
Pericardial effusion	8,840 (2.33)	342 (17.00)	606 (14.51)
Pneumonia	8,347 (2.20)	196 (9.75)	411 (9.84)
Altered mental status	1,821 (0.48)	25 (1.25)	71 (1.72)
Splenic vein thrombosis	986 (0.26)	30 (1.50)	46 (1.11)
Need for vasopressors	417 (0.11)	45 (2.25)	46 (1.11)
Abdominal compartment syndrome	304 (0.08)	20 (1.00)	46 (1.11)
DIC	228 (0.06)	10 (0.5)	20 (0.49)
Pancreatic pseudoaneurysms	38 (0.01)	0 (0.0)	0 (0.0)
Need for Debridement/ duct drainage	5,274 (1.39)	140 (7.00)	174 (4.18)

In the multivariable logistic regression analysis, PN was associated with a higher likelihood of at least any complication compared to EN (aOR: 2.167; 95%CI: 1.801-2.607; P<0.001 and aOR: 2.043; 95%CI: 1.585-2.633; P<0.001), respectively. EN was specifically linked to a higher likelihood of locoregional complications (P=0.045), while PN was associated with systemic complications (P<0.001).

The LOS

The study's overall mean LOS was 4.3 ± 0.2 days. Both the EN and PN cohorts had similar mean LOS values: 16.7 ± 0.7 days and 16 ± 0.5 days, respectively (p=0.547). Discharges without EN or PN had a shorter mean LOS of 4.13 ± 0.2 days. Approximately 6.67% (25,307) of all admissions lasted 15 days or more (prolonged), with 5.3% and 11% receiving EN and PN, respectively. In contrast, only 5.75% (21,460) of admissions without EN or PN (no need for nutritional support) lasted 10 days or more. After adjustments, admissions involving PN lasted on average 3.91 days longer than those without a record of nutritional support (95%CI: 3.088-4.740; P<0.001). Similarly, admissions involving EN lasted on average 4.5 days longer than those without a record of nutritional support (P<0.001).

On multivariable analysis, admissions involving PN and EN had aOR of prolonged hospitalization (>15 days) of 20.445 (95%CI: 16.894-24.742; P<0.001) and 17.155 (95%CI: 13.113-22.444; P<0.001), respectively. Comparatively, admissions without nutritional support had a significantly lower likelihood of prolonged hospital stay (aOR: 0.048; 95%CI: 0.041-0.057; P<0.001). Factors associated with prolonged hospitalization included higher CCI, advanced age, admission to larger hospitals, Black race, the occurrence of any complication, higher APR-DRG severity of illness class, or the need for any procedural intervention (P<0.001).

Home versus non-home discharges

About 27,128 (7.15%) were transfers from other acute care hospitals. The PN cohort had 732 (17.55%) initial admissions from other hospitals, while the EN cohort had 505 (25.13%) such admissions. Routine home discharges accounted for 83.9% (337,674) of discharges, while 16.1% (61,085) were to other acute care facilities, skilled nursing homes, intermediate care, or home health care (Figure 2). Non-home discharges were more common in hospitalizations with EN compared with PN (49.8% and 45.3% respectively) after adjusting for the admission route. EN hospitalizations were more likely to result in non-home discharge (aOR: 2.983; 95%CI: 2.200-4.044; P<0.001) than hospitalizations with PN (aOR: 2.369; 95%CI: 1.975-2.842; P<0.001 respectively).

Prolonged hospitalization, severe AP, advancing age, higher CCI, and complications were associated with higher odds of non-home discharge (P<0.001). Conversely, being female, receiving care at larger hospitals, having insurance coverage, and being of Hispanic or Asian ethnicity were associated with decreased odds of non-home discharge (P<0.001).

## Discussion

The index study contributes useful insights into mortality, complications, LOS, and discharge outcomes in AP patients receiving different modes of nutrition. Current evidence suggests that early EN may be associated with better outcomes and reduced mortality rates compared to PN for acute severe pancreatitis and is recommended by the American College of Gastroenterology as first-line nutrition in patients who cannot maintain normal oral feeding [23].

The index study recorded a total of 2,280 mortalities. After accounting for disease severity, EN was linked to lower odds of mortality, whereas PN remained associated with an increased likelihood of mortality regardless of disease severity. These findings emphasize the importance of considering disease severity when evaluating the impact of nutrition mode on mortality. The results appear to validate the American College of Gastroenterology guidelines for nutritional support in AP patients [23], warranting further investigation and clarification of EN's impact on acute severe pancreatitis outcomes. However, normal oral feeding was associated with better outcomes compared to EN or PN, likely reflecting lower disease severity or comorbidity burden in this population. Normally, oral feeding is preferred when patients can tolerate it without exacerbating symptoms. Overall, the study suggests that EN might be more beneficial than PN in reducing mortality in patients with severe AP. PN has been associated with a higher risk of mortality in AP patients [24,25]. Conversely, EN is reported to reduce mortality across all disease severities [26-28]. Nonetheless, early initiation of EN did not improve mortality odds in this study, suggesting the contribution of other factors beyond the timing of EN initiation in determining mortality outcomes.

In terms of complications, the study found that both EN and PN were significantly associated with an increased likelihood of complications. These findings are consistent with a previous study, reporting higher complication rates in patients receiving artificial nutrition [29]. The association between EN and locoregional complications, and PN with systemic complications in patients with AP can be understood through their distinct routes of administration and physiological effects. EN involves delivering nutrients directly into the gastrointestinal tract, which can stimulate the pancreas and potentially exacerbate local inflammation, leading to locoregional complications. On the other hand, PN bypasses the gastrointestinal tract and directly delivers nutrients into the bloodstream, reducing the direct stimulation of the pancreas. However, this systemic delivery can trigger an inflammatory response, leading to systemic complications. Clinicians often tailor the choice of nutrition mode based on the patient's disease severity and gastrointestinal function to strike an effective balance between providing necessary nutrition for healing and minimizing adverse effects on the inflamed pancreas. However, further research is needed to elucidate the specific mechanisms behind these associations and to optimize nutrition strategies in patients with AP to reduce complications and improve overall outcomes. The index study also observed that patients admitted for AP who received normal oral feeding had significantly lower odds of developing complications, irrespective of disease severity. This finding appears to validate the practice of continuing normal oral feeding in mild and moderately severe pancreatitis.

The index study found that both EN and PN were associated with longer LOS compared to normal oral feeding. The adjusted odds of prolonged hospitalization were significantly higher for admissions involving EN and PN compared to normal oral feeding. These findings may be explained by the duration of nutritional support and the effect of disease severity on the need for prolonged nutritional support and on the LOS. The study revealed a higher likelihood of non-home discharge for hospitalizations involving either EN or PN. However, this association is likely also influenced by the disease severity and duration of nutritional support and is best interpreted within that context. The association of prolonged hospitalization, disease severity, older age, higher comorbidity index, and the presence of complications with an increased likelihood of non-home discharge is consistent with previous studies [30,31]. Conversely, being female, receiving care at larger hospitals, having insurance coverage, and identifying as Hispanic or Asian were associated with reduced odds of non-home discharge, which may reflect various socio-demographic factors and healthcare disparities.

The current study has some strengths that can be appreciated. It draws on data from a large sample representative of a diverse patient population from multiple healthcare centers. With its all-payer design, the NIS encompasses patients from various insurance types, including Medicare, Medicaid, private insurance, and uninsured individuals, providing a comprehensive view of disease burden across different payer categories. However, the study is not without limitations. The reliance on ICD codes in the NIS data limits the ability to infer specific patient selection details and factors related to AP severity without reviewing individual hospitalization charts and physician notes, which are unavailable in the NIS. Consequently, the study may not fully account for the potential bias arising from the likelihood of selecting healthier patients for oral nutrition, leading to better outcomes. Nonetheless, the study adjusts for disease severity and risk of mortality using validated APR-DRG mortality and severity of illness variables from previous research to gain useful insights on this important clinical topic.

## Conclusions

EN demonstrated better outcomes in mortality rates and systemic complications than PN. It should be the preferred initial approach for patients unable to maintain normal oral feeding. A careful selection of the appropriate mode of nutritional support for each AP hospitalization should be prioritized over the timing of nutritional support initiation. There is a need for further research to explore the specific mechanisms that explain the impact of nutrition mode on both locoregional and systemic complications in AP.

## Appendices

**Table 4:** ICD-10 diagnostic and procedure codes used in the study ICD-10, International Classification of Diseases, 10th Edition; GI, gastrointestinal tract

ICD-10 codes	Code description
K85	Acute pancreatitis
3E0336Z	Introduction of nutritional substance into peripheral vein, percutaneous approach
3E0436Z	Introduction of nutritional substance into central vein, percutaneous approach
3E0G36Z	Introduction of nutritional substance into upper GI, percutaneous approach
3E0H36Z	Introduction of nutritional substance into lower GI, percutaneous approach
K862	Pancreatic cyst
K863	Pancreatic pseudocyst
I82.890	Splenic vein thrombosis
J90	Pleural effusion
J80	Acute respiratory distress syndrome
N17.9	Acute kidney failure
3E053XZ, 3E063XZ, 3E033XZ	Introduction of vasopressor into central or peripheral vessel, percutaneous approach
J19.9	Pneumonia
D65	Disseminated intravascular coagulopathy
M79A3	Nontraumatic compartment syndrome of abdomen
R41.82	Altered mental status
0FTG0ZZ, 0FTG4ZZ	Pancreatic resection
0FJB8ZZ	Endoscopic retrograde cholangiopancreatography
